# High-dose steroids in high pain responders undergoing total knee arthroplasty: a randomised double-blind trial

**DOI:** 10.1016/j.bja.2021.10.001

**Published:** 2021-11-05

**Authors:** Niklas I. Nielsen, Henrik Kehlet, Kirill Gromov, Anders Troelsen, Henrik Husted, Claus Varnum, Per Kjærsgaard-Andersen, Lasse E. Rasmussen, Lina Pleckaitiene, Nicolai B. Foss

**Affiliations:** 1 Department of Anaesthesiology, Copenhagen University, Hvidovre Hospital, Copenhagen, Denmark; 2 Section of Surgical Pathophysiology, Rigshospitalet, University of Copenhagen, Copenhagen, Denmark; 3 Department of Orthopaedic Surgery, Copenhagen University, Hvidovre Hospital, Copenhagen, Denmark; 4 Department of Orthopaedic Surgery, Lillebaelt Hospital, Vejle, Denmark; 5 Department of Anaesthesiology, Lillebaelt Hospital, Vejle, Denmark

**Keywords:** anaesthesia, dexamethasone, fast-track surgery, high-dose steroids, high pain responders, multimodal analgesia, postoperative pain, total knee arthroplasty

## Abstract

**Background:**

Total knee arthroplasty (TKA) is associated with moderate-to-severe postoperative pain despite multimodal opioid-sparing analgesia. Pain catastrophising or preoperative opioid therapy is associated with increased postoperative pain. Preoperative glucocorticoid improves pain after TKA, but dose-finding studies and benefit in high pain responders are lacking.

**Methods:**

A randomised double-blind controlled trial with preoperative high-dose intravenous dexamethasone 1 mg kg^−1^ or intermediate-dose dexamethasone 0.3 mg kg^−1^ in 88 patients undergoing TKA with preoperative pain catastrophising score >20 or regular opioid use was designed. The primary outcome was the proportion of patients experiencing moderate-to-severe pain (VAS >30) during a 5 m walk 24 h postoperatively. Secondary outcomes included pain at rest during nights and at passive leg raise, C-reactive protein, opioid use, quality of sleep, Quality of Recovery-15 and Opioid-Related Symptom Distress Scale, readmission, and complications.

**Results:**

Moderate-to-severe pain when walking 24 h postoperatively was reduced (high dose *vs* intermediate dose, 49% *vs* 79%; *P*<0.01), along with pain at leg raise at 24 and 48 h (14% *vs* 29%, *P*=0.02 and 12% *vs* 31%, *P*=0.03, respectively). C-reactive protein was reduced in the high-dose group at both 24 and 48 h (both *P*<0.01). Quality of Recovery-15 was also improved (*P*<0.01).

**Conclusions:**

When compared with preoperative dexamethasone 0.3 mg kg^−1^ i.v., dexamethasone 1 mg kg^−1^ reduced moderate-to-severe pain 24 h after TKA and improved recovery in high pain responders without apparent side-effects.

**Clinical trial registration:**

NCT03763734.

**Editor's key points**

• Moderate-to-severe pain is common after knee arthroplasty; this limits early mobilisation and range of joint movements.
• A sizeable proportion of patients undergoing joint arthroplasty have known risk factors for severe pain; these typically have very poor recovery and functional outcomes.
• This trial found that a relatively high dose of i.v. dexamethasone, 1.0 mg kg^−1^, improved analgesic and functional outcomes, and quality of recovery, after knee arthroplasty surgery.

Primary total knee arthroplasty (TKA) is a common surgical procedure and is expected to increase in frequency.^1^, ^2^, ^3^ In spite of extensive research, pain is still a significant problem, both in the early and late recovery after TKA,^4^, ^5^, ^6^ which, combined with fatigue and muscle weakness, inhibits early mobilisation and recovery.^7^ Current multimodal opioid-sparing techniques, including the use of paracetamol, NSAIDs, or cyclooxygenase-2 inhibitors; local infiltration analgesia (LIA); and peripheral nerve blocks (PNBs), still leave many patients with moderate-to-severe postoperative pain.^8^ In addition, specific patient groups are at increased risk of excessive postoperative pain, such as pain catastrophisers and patients on preoperative opioid treatment.^9^, ^10^, ^11^

In recent years, several studies have shown an additional analgesic benefit of perioperative glucocorticoids, often in doses (∼24 mg dexamethasone [DXM] or equivalents) beyond the conventional DXM 4–8 mg dose for postoperative nausea and vomiting prophylaxis.^5^ However, the potential additive effect of higher doses of glucocorticoids has not yet been investigated in TKA.^12^ Research into specific postoperative pain management strategies for risk groups (possible high pain responders [HPRs]) is limited, and with the ongoing opioid crisis in healthcare worldwide,^13^ it is especially important to investigate personalised opioid-sparing care in HPR patients.

Consequently, the aim of the present study was to perform a clinical trial of high-dose (HD) DXM 1 mg kg^−1^ i.v. *vs* intermediate-dose (ID) DXM 0.3 mg kg^−1^ i.v. administered preoperatively on postoperative pain and early recovery after TKA in patients predicted to be HPRs.

## Methods

This double-blind RCT was designed^14^ to compare intravenous HD DXM 1.0 mg kg^−1^ with an ID DXM 0.3 mg kg^−1^.^5^ The study was done at Hvidovre and Vejle Hospitals in Denmark; approved by the local ethics committee (H-18034778), the Centre for Data Protection (VD-2019-04), and the Danish Medicines Agency (DKMA) EudraCT-number 2018-002635-23; and registered at ClinicalTrials.gov (NCT03763734, first registered on November 28, 2018). Written informed consent was obtained from all subjects before entering the study.

Dexamethasone was administered as a single i.v. bolus dose preoperatively immediately after spinal anaesthesia. Dexamethasone dose (HD or ID) was blinded upon arrival at the operating theatre, and all personnel involved in treating the participants were blinded to allocation. Surgery was performed in standardised fast-track settings, including pre- and postoperative tranexamic acid, LIA, postoperative compression bandage, absence of drains, and the use of a standardised mobilisation protocol encouraging early mobilisation. Perioperative antibiotic regimen consisted of i.v. dicloxacillin 2 g at start of surgery and repeated if body weight >80 kg or if length of surgery >2 h. Dicloxacillin was repeated as 1 g tablet 8 h after end of surgery. Cefuroxime replaced dicloxacillin in patients with allergy.

Surgical procedure included midline incision, medial parapatellar arthrotomy, using measured resection technique with release as needed, osteotomy with extramedullary guide on tibia, intramedullary guide on femur, and patellar resurfacing in all cases at the first centre and in selected cases in the second centre. No computer-aided navigation was used. Femoral tourniquet was used optionally at one site. Patients were discharged to their homes when fulfilling the standard functional discharge criteria. In-hospital only thromboprophylaxis was used in patients with length of stay (LOS) <5 days.

All subjects underwent surgery in planned spinal anaesthesia with hyperbaric bupivacaine 0.5% (10–12.5 mg) and perioperative sedation with propofol optional. Multimodal analgesic standard regimen was initiated on day of surgery with paracetamol 1 g and celecoxib 400 mg, and continued postoperatively with paracetamol 1 g (6 hourly)^−1^, celecoxib 200 mg (12 h)^−1^, and oral morphine 5–10 mg or other opioids in equivalent doses as rescue opioids.^5^ Peripheral nerve blocks were not used.

From January 2019 to August 2020, all patients undergoing elective unilateral primary TKA were screened for inclusion. Patients with Pain Catastrophizing Scale (PCS) score^10^ >20 or a daily opioid intake ≥30 mg of oral morphine or other oral equivalents (for at least 21 days leading up to surgery) were considered HPR and offered inclusion. All participants had to understand, read, and write Danish; be aged 40–90 yr; and give written informed consent. Exclusion criteria included systemic glucocorticoid treatment, neurological damage blurring pain sensation from the surgical area, insulin-dependent diabetes, glucocorticoid allergy, pregnancy/breastfeeding, and ongoing antipsychotic treatment or history of bipolar and schizophrenic disorders.

Subject characteristics data included prior opioid treatment and PCS score, evaluating the level of or tendency to catastrophising thinking,^10^ along with baseline VAS (0–100 mm; 0 mm=no pain and 100 mm=worst possible pain) of pain at rest, at night, and at 5 m walk test. Quality of sleep before and after surgery (numerical rating scale, NRS 0–10; 0=no problem sleeping and 10=worst possible sleep), and pre- and postoperative 24 and 48 h levels of C-reactive protein (CRP) were registered. Postoperative assessments included pain at rest, at 5 m walk test, at passive leg raise, and at night, evaluated with the aforementioned VAS score (0–100 mm; 0 mm=no pain and 100 mm=worst possible pain), at arrival in PACU or the specialised ward, and 4, 24, and 48 h after end of surgery. If discharged before the 48 h assessment, a clinical outpatient follow-up was done. Postoperative recovery was assessed by Quality of Recovery-15 scale (QoR-15)^15^ as total score and subgroup analysis, and Opioid-Related Symptom Distress Scale (OR-SDS)^16^ as composite clinically meaningful event (CME) and number of patients reporting CME, along with quality of sleep.

A pain dairy was completed by the patient on Days 2–7 reporting VAS pain at rest, night, and 5 m walk test, along with quality of sleep, fatigue, nausea, and dizziness all on NRS (0–10; 0=no problem and 10=worst possible). Analgesics and anti-emetics use and overall satisfaction (NRS 0–10; 0=best possible score and 10=worst possible score) were also reported. Follow-up was performed at 14, 30, and 90 days using the electronic patient record or by phone. The Clavien–Dindo^17^ classification was used to grade complications, and all complications were evaluated in relation to index surgery. All data were registered in case-report forms and entered into the REDCap^18^ electronic data capture tool (Vanderbilt University, Nashville, TN, USA).

The primary outcome was the proportion of subjects reporting moderate-to-severe pain (defined as VAS >30) on a 5 m walk test 24 h postoperatively. Secondary outcomes were proportion of patients reporting moderate-to-severe pain (VAS >30) at passive leg raise, at rest, and at night; cumulated pain (cumulated pain was reported VAS scores added from Days 0–2 and 2–7); use of rescue opioids and anti-emetics; quality of sleep 0–48 h and Days 0–7; CRP before and at 24 and 48 h postoperative; QoR-15 and OR-SDS at 0–48 h; quality of sleep, fatigue, nausea, and dizziness on Days 2–7; and overall satisfaction with analgesic treatment. Also included was hospital LOS, readmissions, medical events and complications, and 90 day mortality.

No specific data exist on the pain scores of HPR patients undergoing TKA with ID glucocorticoids. Previous studies have found a prevalence of VAS >30 at 24 h walk test of 0.9 in patients with high PCS, and 0.86 in patients with chronic preoperative opioid use, without preoperative glucocorticoid treatment.^9 11^ We estimated a relative reduction in pain from the ID of glucocorticoids, similar to that found in a previous study,^5^ correlating with an incidence of VAS >30 of 0.7, which was used for power calculation. To detect a 50% reduction in proportion of patients experiencing VAS >30 upon 5 m walk test 24 h postoperatively in a superiority design, using two-sided level of significance 0.05 with a power of 90% required 41 patients in each arm, and we planned inclusion of 44 patients in each arm to allow for dropouts (*n*=88).

A computer-generated randomisation protocol of 88 planned participants, 44 in each group HD *vs* ID, with an 8:8 allocation rate, was packed in 88 opaque envelopes by two unblinded personnel not otherwise participating in the study. Screening and inclusion were done by blinded primary investigators and study personnel, and included participants were consecutively assigned the next randomisation envelope on-site. All participants were also blinded. Randomisation envelopes were opened by unblinded personnel when preparing the study drug ahead of surgery and sealed again afterwards for storage until all study analyses were completed. The study drug was mixed in a saline container to a total volume of 100 ml, and given to the patient after administration of spinal anaesthesia. The 100 ml containing the study drug was blinded for all personnel and participants.

The VAS scores were reported as median and inter-quartile range. Continuous data were tested using *t*-test and presented as means and standard deviations, or Mann–Whitney *U*-test if appropriate. We used SPSS statistics version 25 (IBM, Armonk, NY, USA) and RStudio version 1.0.153 (RStudio, Boston, MA, USA). All data were analysed before unblinding.

## Results

**Fig 1:**
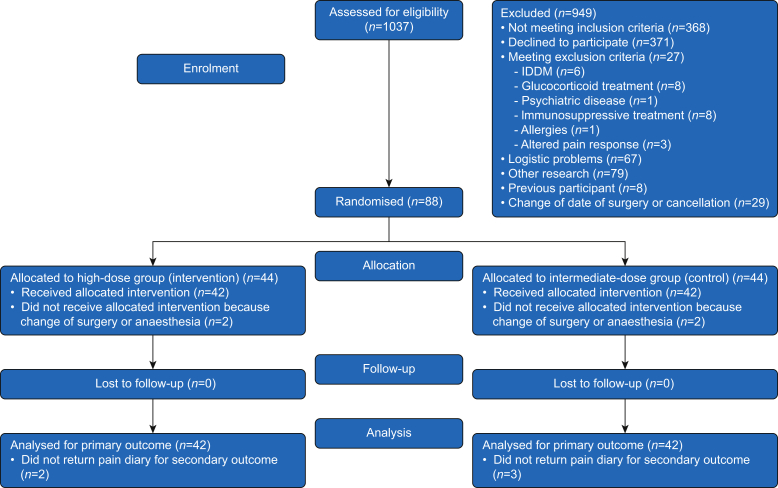
Consolidated Standards of Reporting Trials flow diagram. IDDM, insulin-dependent diabetes mellitus.

From February 13, 2019 to August 26, 2020, a total of 1037 patients were assessed for inclusion, and 88 patients were included and randomised. Four patients did not receive the allocated intervention because of preoperative change of type of surgery or anaesthesia (see Consolidated Standards of Reporting Trials in Fig. 1). Of 1037 patients, 766 either did not meet the inclusion criteria, met the exclusion criteria, or declined participation, and thus, 271 patients (26% of all screened patients) were assessed as HPR patients and considered for inclusion. In addition, 183 patients were not included because of logistical problems, participation in other research, previous participation in our study, or change of date of surgery. All patients included in the study had spinal anaesthesia with or without perioperative sedation, and all patients who received the intervention completed the primary outcome. Five subjects in total, two from the HD group and three from the ID group, did not return the patient diary after Day 7. The baseline characteristics are shown in Table 1.

**Table 1:** Baseline subject characteristics. Characteristics presented as n, median (IQR) or per cent. CRP, C-reactive protein; DASI, Duke Activity Status Index; HD, high-dose group; ID, intermediate-dose group; IQR, inter-quartile range; LIA, local infiltration analgesia; PCS, Pain Catastrophizing Scale. Quality of sleep on a 10-point Likert scale (0=no problems and 10=worst possible sleep). PCS score (inclusion criteria >20; 13-item questionnaire 0–4; maximum score 52). ∗Preoperative opioid therapy: ≥30 mg daily of oral morphine or oral equivalents for at least 21 days leading up to surgery. ^†^Median dose of preoperative opioid in milligrams of morphine (range) in patients included with this criterion. ^‡^All patients were offered APAP and NSAID as preoperative analgesia in the morning of surgery, but 12% vs 15% in HD vs ID group only administered APAP. ^¶^All patients were offered propofol sedation, but some declined and had no sedatives during surgery.

Characteristics	HD (*n*=42)	ID (*n*=42)
Age (yr), median (range)	70 (50–86)	70 (50–82)
Sex (female, %)	66	66
Body mass index (kg m^−2^)	30 (28–37)	31 (27–36)
American Society of Anesthesiologists physical status (1/2/3)	5/20/17	5/31/6
DASI score	29 (19–37)	29 (23–37)
Preoperative data		
Pain reported as VAS >30		
At rest, *n* (%)	14 (33)	13 (31)
5 m walk test, *n* (%)	28 (67)	23 (55)
At night, *n* (%)	21 (50)	22 (52)
Quality of sleep 0–10, median (IQR)	5 (2–8)	5 (3–7)
Pain Catastrophizing Scale, median (IQR)	29 (26–35)	30.5 (24–38)
Number of subjects with preoperative opioid therapy∗	3	4
Median dose (mg) (range)^†^	40 (40–40)	42.5 (40–50)
CRP (mg L^−1^), median (IQR)	3 (1–6)	2 (1–6)
Preoperative both paracetamol and NSAID, *n* (%)^‡^	37 (88)	36 (85)
Perioperative data		
Duration of surgery (min), median (range)	69 (45–103)	68 (44–110)
Propofol sedation (yes/no)^¶^	38/4	35/7
Spinal bupivacaine (mg)	10 (10–12)	10 (10–12)
Use of femoral tourniquet (yes/no)	9/33	8/34
Local infiltration analgesia (mg)	300 (300–400)	300 (300–400)
Intraoperative bleeding (ml)	160 (50–300)	165 (100–250)

### Primary outcome

**Fig 2:**
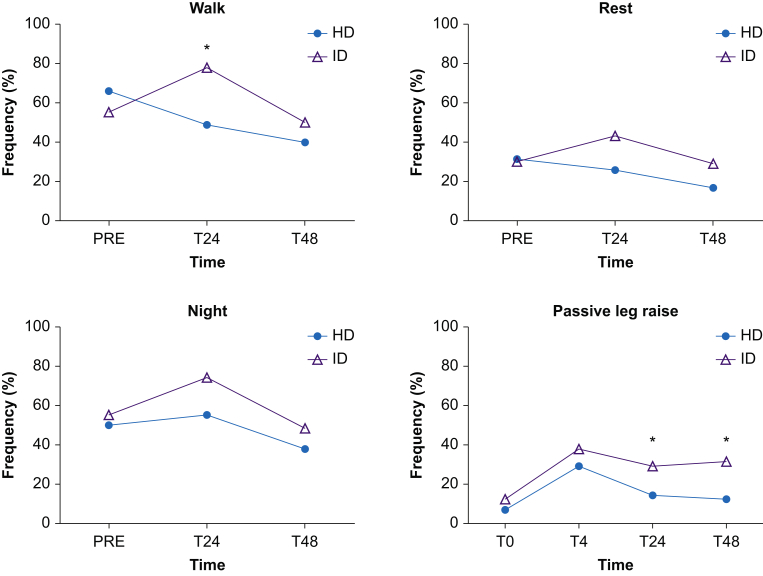
Proportion of VAS >30 in a 5 m walk test, at rest, at night, and in passive leg raise. HD, high-dose group; ID, intermediate-dose group; PRE, preoperatively; T0, at end of surgery; T4, 4 h after end of surgery; T24, 24 h after end of surgery; T48, 48 h after end of surgery. ∗Significant difference (χ^2^ test).

**Fig 3:**
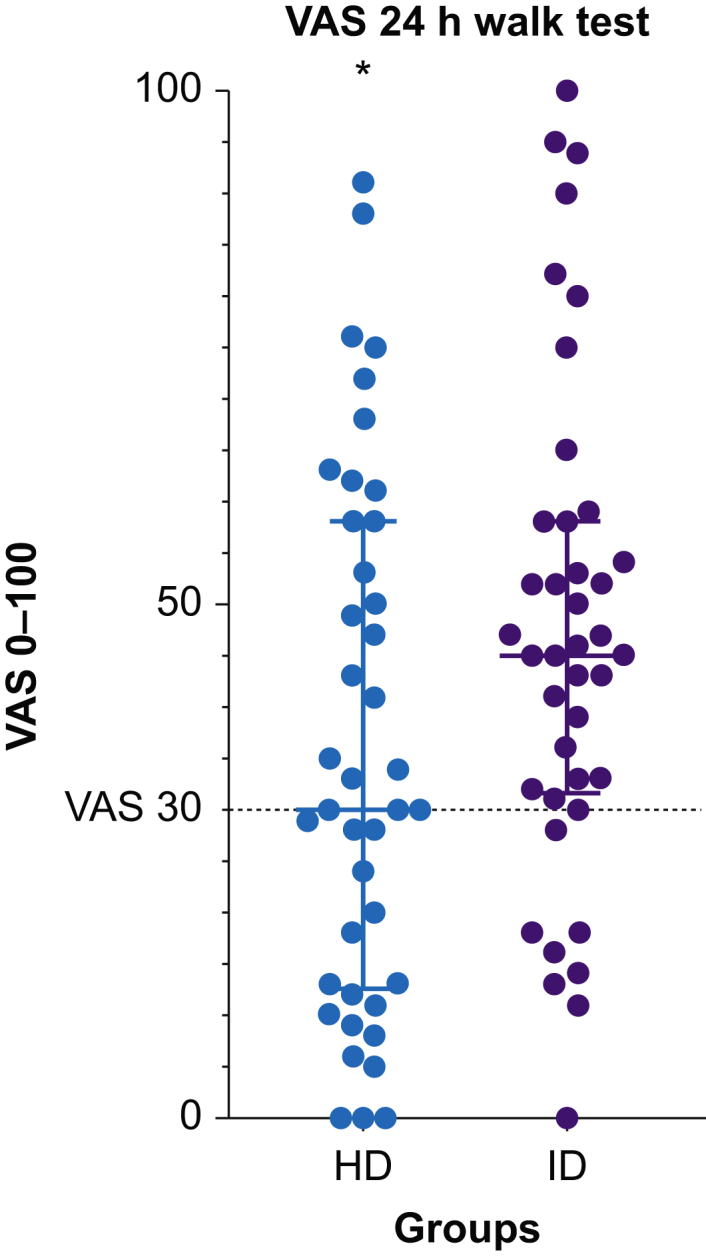
Distribution of VAS 24 h after surgery in a 5 m walk test. HD, high-dose group; ID, intermediate-dose group. Solid line represents median and whiskers inter-quartile range. Dotted line at VAS 30 (primary outcome). ∗Statistically significant difference between groups (Mann–Whitney *U*-test).

The proportion of subjects reporting moderate-to-severe pain (VAS >30) 24 h after surgery was significantly lower in the HD group compared with the ID group in a 5 m walk test (49% *vs* 79%; *P*<0.01) (Fig. 2), and VAS scores were median 30 [13–58] *vs* 45 [32–58]; *P*=0.05 (Fig. 3, for distribution of pain scores at 24 h). All but one patient from each group were able to complete the 5 m walk test at 24 h, and both reported VAS >30 in trying to mobilise.

### Secondary outcomes

The proportion of subjects reporting moderate-to-severe pain (VAS >30) with passive leg raise was lower in HD *vs* ID (14% *vs* 29%; *P*=0.02 and 12% *vs* 31%; *P*=0.03) at both 24 and 48 h, respectively. No significant difference was found at 5 m walk test at 48 h and at rest or at sleep (see Fig. 2). From Days 2–7, a significant difference in proportion of patients reporting VAS >30 was found in 5 m walk test and at rest on Days 4–5, but not at night (Supplementary Appendix 1).

Cumulated pain Days 0–2 showed a difference at rest (HD 80 [37–112] *vs* ID 86 [69–143]; *P*=0.05, and at passive leg raise HD 23 [0–81] *vs* ID 67 [28–11]; *P*=0.05, but no difference at 5 m walk test (HD 82 [45–118] *vs* ID 101 [69–131]; *P*=0.06), or at night (HD 72 [24–100] *vs* ID 90 [56–109]; *P*=0.07). No difference was found in cumulated pain over Days 2–7 at rest, at night, or at 5 m walk test.

**Fig 4:**
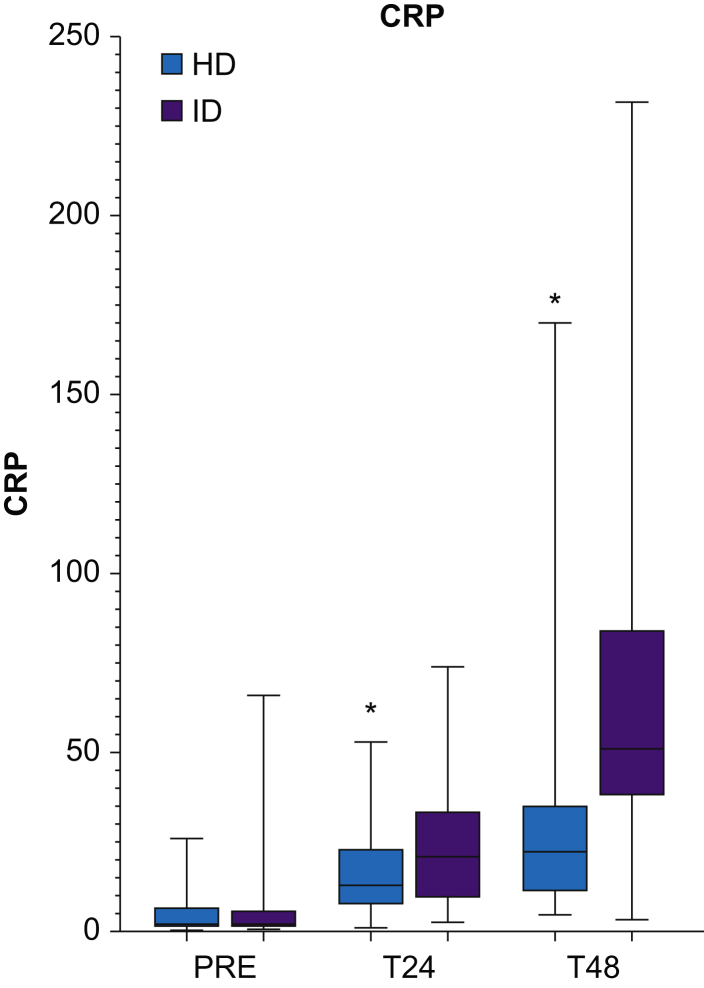
Changes in C-reactive protein (CRP) from preoperatively (PRE), 24 h (T24), and 48 h (T48) after end of surgery. Boxes represent inter-quartile range; line is median and whiskers min–max. ∗Statistical significance (Mann–Whitney *U*-test).

Inflammatory response measured by CRP was significantly lower in the HD group both at 24 h (13 [8–23] mg L^−1^ *vs* 21 [9–34] mg L^−1^; *P*=0.01) and 48 h (23 [12–35] mg L^−1^ *vs* 51 [39–83] mg L^−1^; *P*<0.01) (Fig. 4).

Cumulated use of opioids (presented as oral morphine, mg) on Days 0–2 showed no significant difference between HD and ID (median 51 [30–90] *vs* 83 [46–111]; *P*=0.06) (Supplementary Appendix 2), nor cumulated opioid use on Days 2–7 (in oral mg of morphine) (median 95 [18–185] *vs* 150 [45–255]; *P*=0.12).

Ondansetron was the only used anti-emetic, but without difference between the groups HD *vs* ID. Cumulated anti-emetic use in the HD *vs* ID groups at 48 h was 8 mg *vs* 36 mg (*P*=0.21), and the incidence of subjects in need of anti-emetic at 24 h was (7 [16] *vs* 5 [12]; *P*=0.75) and at 48 h was (1 [2] *vs* 8 [19]; *P*=0.02).

Total number of reported CME in OR-SDS was similar between groups (Supplementary Appendix 3). Total score of the QoR-15 (range 0–150) was higher in HD *vs* ID at 48 h (median 130 [124–140] *vs* 122 [108–133]; *P*=0.01). Also, delta value of total score from preoperatively to 24 h was higher in the HD group (5 [–6 to 13] *vs* –9 [–23 to 7]; *P*=0.03), and from preoperatively to 48 h (15 [4–23] *vs* –1 [–15 to 12]; *P*<0.01). Subgroups 1, 4, and 5 had significantly higher scores at 48 h (*P*=0.02, *P*=0.03, and *P*<0.01) in favour of HD (Table 2).

**Table 2:** Quality of Recovery-15 (QoR-15) presented as median (IQR). HD, high-dose group (*n*=42); ID, intermediate-dose group (*n*=42); T4, 4 h after end of surgery; T24, 24 h after end of surgery; T48, 48 h after end of surgery; T4-PRE, delta values presenting the change from before and onto 4 h after end of surgery; T24-PRE, delta values presenting the change from before and onto 24 h after end of surgery; T48-PRE, delta values presenting the change from before and onto 48 h after end of surgery.

QoR-15, median (IQR)	Score range	Preoperatively		T4			T24			T48		
Subgroups		HD	ID	HD	ID	*P*-value	HD	ID	*P*-value	HD	ID	*P*-value
1. Physical comfort	0–50	44 (39–46)	45 (40–49)	40 (35–42)	40 (35–44)	0.42	39 (31–46)	42 (35–46)	0.32	47 (42–50)	44 (39–48)	0.02
2. Physical independence	0–20	19 (16–20)	19 (16–20)	7 (0–13)	8 (1–11)	0.88	15 (10–18)	12 (10–16)	0.23	15 (11–19)	13 (11–17)	0.19
3. Psychological support	0–20	20 (20–20)	20 (20–20)	20 (20–20)	20 (20–20)	0.83	20 (20–20)	20 (20–20)	0.57	20 (20–20)	20 (20–20)	0.13
4. Pain	0–20	7 (4–10)	8 (5–13)	11 (8–18)	10 (7–16)	0.49	10 (5–15)	7 (4–10)	0.05	12 (8–16)	9 (7–14)	0.03
5. Emotional state	0–40	29 (23–35)	31 (23–38)	38 (32–40)	35 (30–40)	0.36	40 (34–40)	36 (29–40)	0.09	40 (38–40)	38 (28–40)	<0.01
Total questionnaire score	0–150	114 (105–128)	124 (103–133)	113 (103–123)	115 (102–121)	0.94	119 (106–130)	112 (102–125)	0.19	130 (124–140)	122 (108–133)	<0.01

Delta QoR-15	Score range	T4-PRE			T24-PRE			T48-PRE		
Subgroups		HD	ID	*P*-value	HD	ID	*P*-value	HD	ID	*P*-value
1. Physical comfort	0–50	–3 (–10 to 1)	–5 (–10 to 3)	0.75	–4 (–10 to 2)	–3.5 (–10 to 2)	0.96	2 (–1 to 7)	0 (–5 to 3)	0.04
2. Physical independence	0–20	–10 (–16 to –5)	–11 (–16 to –6)	0.71	–2 (–5 to 0)	–5 (–10 to –1)	0.06	–2 (–4 to 0)	–5 (–8 to 1)	0.15
3. Psychological support	0–20	0 (0–0)	0 (0–0)	0.68	0 (0–0)	0 (0–0)	0.98	0 (0–0)	0 (0–0)	0.62
4. Pain	0–20	4 (0–11)	3 (–2 to 8)	0.28	1 (–2 to 5)	–1 (–6 to 5)	0.03	2 (0–8)	3 (–4 to 6)	0.06
5. Emotional state	0–40	6 (0–15)	2 (–1 to 9)	0.09	7 (2–13)	2 (–2 to 10)	0.06	8 (2–15)	2 (–2 to 10)	0.01
Total questionnaire score	0–150	–1 (–19 to 8)	–12 (–22 to 2)	0.15	5 (–6 to 13)	–9 (–23 to 7)	0.03	15 (4–23)	–1 (–15 to 12)	<0.01

There was no difference in quality of sleep between the groups before or after surgery on Days 0–7, and neither fatigue, nausea, nor dizziness reported in the pain diary showed difference between the groups on Days 2–7 (Supplementary Appendix 4).

Overall satisfaction with analgesic treatment (Days 0–7) was similar between the groups (*P*=0.64). Length of stay was similar, *P*=0.44, with only one *vs* two subjects having LOS >2 days. One subject in the HD group *vs* two in the ID group were readmitted to the hospital within the first week after surgery, because of suspected prosthetic joint infection (not verified) and problems with pain and mobilisation. Medical complications at 90 days in the two groups consisted of two skin infections (herpes zoster and erysipelas) in the HD group and one subclavian vein thrombosis in the ID group. Surgical complications included one joint infection demanding revision surgery, one wound leakage stopped by compression bandages, and one manipulation under anaesthesia in the HD group *vs* one deep tissue infection needing antibiotics and one patellar dislocation needing revision surgery in the ID group. Clavien–Dindo grade (I/II/IIIb) in the HD group *vs* the ID group was 1/0/2 *vs* 1/0/1.

## Discussion

This is the first study to compare different high doses (1.0 *vs* 0.3 mg kg^−1^) of dexamethasone in TKA and the first to specifically study an high pain responder population. The incidence of moderate-to-severe pain (primary outcome) was significantly reduced using high-dose dexamethasone i.v.

Earlier cohort studies identified anxiety, depression, trait anxiety, and preoperative opioid use (HPR) as factors associated with persistent pain and prolonged postoperative opioid use after TKA.^19^^,^^20^ In this study, the incidence of HPR patients was 26% of all screened patients, highlighting the importance of evaluating pain management strategies specifically in these patients. High pain responder patients have high pain levels postoperatively, despite multimodal analgesic therapy. As such, we found an incidence of moderate-to-severe pain of 79%, with optimised multimodal analgesia, including LIA and ID glucocorticoid comparative with previous findings of 90% in HPR patients with multimodal analgesia without glucocorticoids.^11^

The incidence of moderate-to-severe pain was reduced to 49% using HD DXM, which is still high and calls for a more specialised approach in treating the HPR population. Earlier studies of specific interventions in HPR patients were limited to one study, excluding patients who receive opioid medication, showing antidepressants (selective serotonin reuptake inhibitors) to have no or limited effect on pain^11^ and psychological intervention studies with cognitive behavioural therapy, which had very limited effects.^21^

The demonstrated additional analgesic effect in the HD group was correlated with a further attenuated CRP response 24 and 48 h after surgery compared with ID, similar to the relative effects seen when comparing an ID glucocorticoid dose with placebo in TKA.^5^ The anti-inflammatory effect of DXM had wained after 48 h as expected from the pharmacodynamic profile, accompanied by an increase in pain on the evening of Day 2 in the HD group (40% at 48 h; 53% on Day 2 in the evening), bringing the proportion of patients in HD to the same level as ID. This suggests a potential beneficial effect of a repeated DXM dose on Day 1 or 2 after surgery to prolong the attenuating effect on pain and the inflammatory response to surgery. Earlier studies on repeated doses have shown some effects on postoperative pain within the first 48 h,^22^, ^23^, ^24^ but in these studies, the preoperative dose was intermediate (DXM 4–20 mg) compared with the HD used in the present study.

The QoR-15 showed statistically significant differences, favouring the HD group. This could be explained by the analgesic effect and reduced inflammation facilitating decreased fatigue, as QoR-15 showed significant improvements in physical comfort and emotional state subgroups, but psychotropic effects of glucocorticoids may have contributed to the physical well-being.

Cumulated postoperative use of rescue opioids from surgery to 48 h did not achieve statistical significance between the two groups, although a tendency towards a lower use was seen in the HD group, which could be a Type 2 error, as the study was not powered to show differences in opioid consumption.

In a recent large systematic review on postoperative pain treatment in TKA,^25^ the dose of postoperative opioids was comparable with our findings, in spite of our study including HPR patients exclusively.

The HPR group might be at higher risk of both continued prescription of opioids during their recovery and subsequently at higher risk of opioid addiction as a consequence of their higher postoperative pain levels, especially for patients with preoperative opioid use.^13^ Reduction of postoperative pain by non-opioid strategies is recognised as an important tool in preventing postoperative opioid abuse,^26^ and the improved pain management by non-opioid strategies presented by HD in HPR constitutes yet an important perioperative asset in preventing opioid use/abuse after surgery. It is notable from our data that the incidence of preoperative opioid prescription in the recruited group was low and the compliance with prescription of preoperative non-opioid analgesics was high compared with previously reported data,^27^ which could be a consequence of recruitment bias, but could also be a result of good institutional/national practices. Nonetheless, the HPR group is still at high risk of inadequate pain management and high levels of postoperative pain,^26^ and development of effective non-opioid strategies, including nerve blocks, is an important research issue in this patient group.^28^

In HD glucocorticoid treatment, potential side-effects need consideration. Previous studies found no increased risk of infections with ID (DXM 0.3 mg kg^−1^ equivalents).^6^ Safety studies^29^^,^^30^ found no issues in using glucocorticoids in TKA nor prosthetic joint infection or other complications, and although no safety studies exist on HD DXM specifically in orthopaedic surgery, HD DXM had no safety issues in cardiac surgery.^31^ The number of joint infections in our study showed no difference between the ID and HD groups, but our study was inadequately powered in regard to detecting this rare outcome.

No patients had symptoms of delirium, and all were able to fill in questionnaires on Days 0–2, consistent with a very low incidence of clinically relevant postoperative delirium symptoms within enhanced recovery programmes.^32^ The use of perioperative steroids might further contribute to this low incidence.^33^^,^^34^ Sleep quality is important in the postoperative phase,^35^ and may be associated with pain, opioid use, and inflammation, but no difference was seen between the groups in our study.

A strength of this study is the specialised arthroplasty units having similar fast-track set-ups for many years,^7^ whilst limitations include the use of self-reported pain and the pain diary after 48 h. External validity was supported by the internationally accepted evidence-based fast-track set-up,^28^ opioid-sparing multimodal analgesia regimen with neuraxial block, and infiltration analgesia. However, this is also a limitation when interpreting effects in other set-ups and regimes, including the use of PNB, although HD might rationally be expected to decrease postoperative pain and opioid consumption even more in regimes without multimodal analgesia. As such, the use of other well-documented peri- and postoperative improvements in our fast-track set-up might minimise the effect of HD DXM.

Another limitation lies in the use of the HPR group itself and the willingness of this group to participate in an RCT. We do not know if declining participation is more common in this patient group, which precludes interpretation of selection bias. The absence of long-term follow-up on pain, opioid use, and physical and psychological well-being is another limitation, but probably non-existent based upon the used detailed 1 week follow-up.

In conclusion, an high dose of preoperative steroids (DXM 1 mg kg^−1^ i.v.) led to a significant reduction in patients with moderate-to-severe pain 24 h after surgery, compared with intermediate dose (DXM 0.3 mg kg^−1^ i.v.), and reduced CRP response together with improved quality of recovery. However, a clinical pain problem still resides in this high pain responder population, as 50% of patients registered VAS >30 during a brief walk at 24 h after surgery.

## Authors' contributions

Drafting of protocol: NIN, HK, HH, KG, NBF

Writing of paper: all authors

Approval of paper: all authors.
