# Supplementary material for: High-dose steroids in high pain responders undergoing total knee arthroplasty: a randomised double-blind trial
Source: Br J Anaesth. 2021 Nov 5;128(1):150–8. doi: 10.1016/j.bja.2021.10.001 (PMC8787770; doi:10.1016/j.bja.2021.10.001)
Supplement: Multimedia component 1 [file mmc1.docx]

Appendix 1:

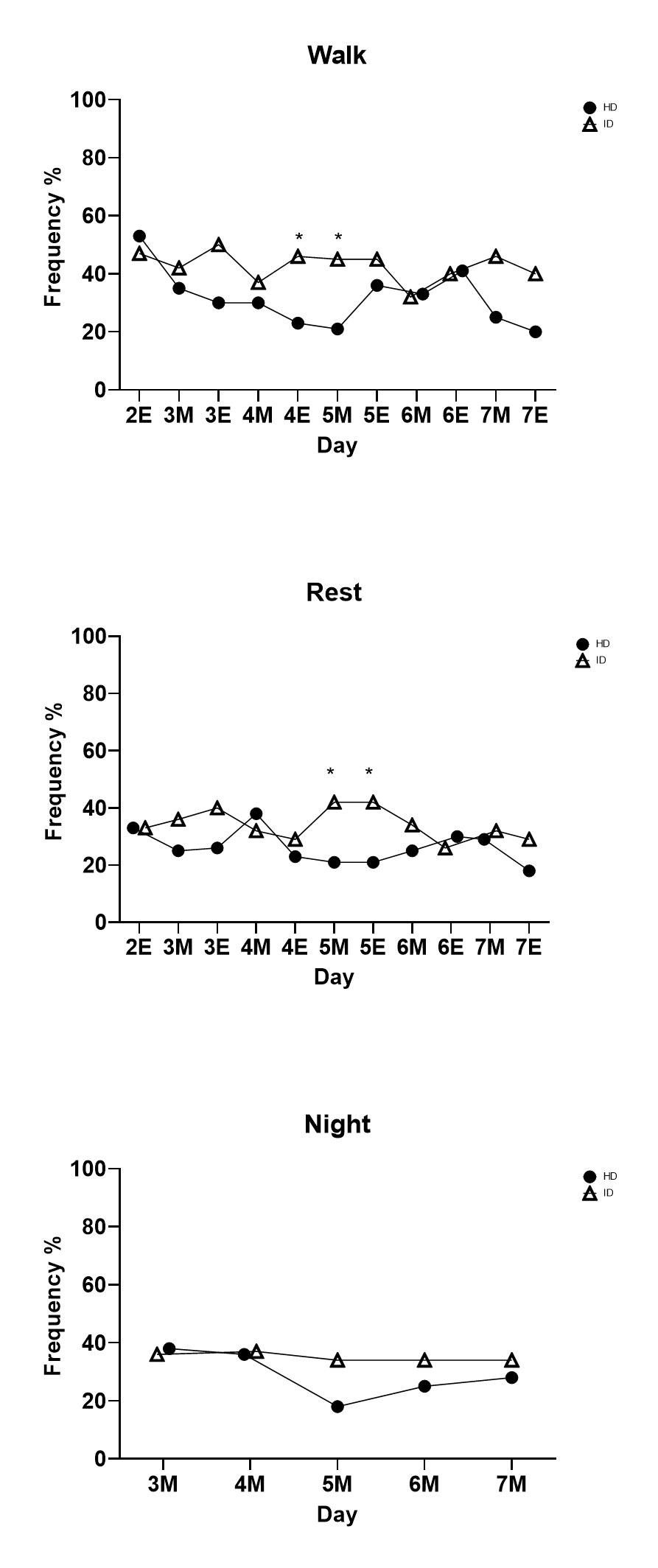

Appendix 1: Frequency of VAS>30 from day 2-7 at rest, walk (5-meter walk test) and at night. HD: High dose group, ID: Intermediate dose group. E: Evening, M: Morning, *indicates statistically significant difference (chi-squared test).

Appendix 2

|  | **HD** median (IQR) | **ID** median (IQR) | ***p*** *(Mann-Whitney test)* |
| --- | --- | --- | --- |
| **T0** | 0 (0-0) | 0 (0-0) | *0.86* |
| **T24** | 30 (20-53) | 50 (23-70) | *0.08* |
| **T48** | 15 (0-30) | 25 (10-45) | *0.07* |
| **Cumulated day 0-2** | 51 (30-90) | 83 (46-111) | *0.06* |

Appendix 2:
Opioid-use represented as oral morphine (mg) from day 0 onto day 2, and cumulated day 0-2 (median (IQR). HD: High dose group, ID: Intermediate dose group, T0: Opioids given in the post anaesthesia care unit, T24: opioid administered from last registration T0 and onto 24h after end of surgery, T48: opioid administered from last registration T24 and onto 48h after end of surgery. Significance-test applied: Mann-Whitney test.

Appendix 3:

| Composite CME | HD | ID | *p-value* |
| --- | --- | --- | --- |
| T4 (n) | 14 | 10 | *0.73* |
| T24 (n) | 15 | 23 | *0.35* |
| T48 (n) | 5 | 22 | *0.13* |
| Cumulated (n) | 34 | 55 | *0.27* |
| Number of patients experiencing CME | HD | ID | *p-value* |
| T4 n (%) | 8 (19%) | 6 (14%) | *0.56* |
| T24 n (%) | 7 (17%) | 12 (29%) | *0.19* |
| T48 n (%) | 4 (10%) | 12 (29%) | ***0.03*** |
| Overall from day 0-2 n (%) | 13 (31%) | 17 (41%) | *0.32* |

Appendix 3:
Clinically meaningful event (CME) in the OR-SDS. Composite CME is the number of CME’s at given timepoints 4h, 24h, 48h and cumulated, and number of patients experiencing a CME at time points 4h, 24h, 48h and overall, from day 0-2. **Bold** indicates statistical significance (Chi-squared test).

Appendix 4:

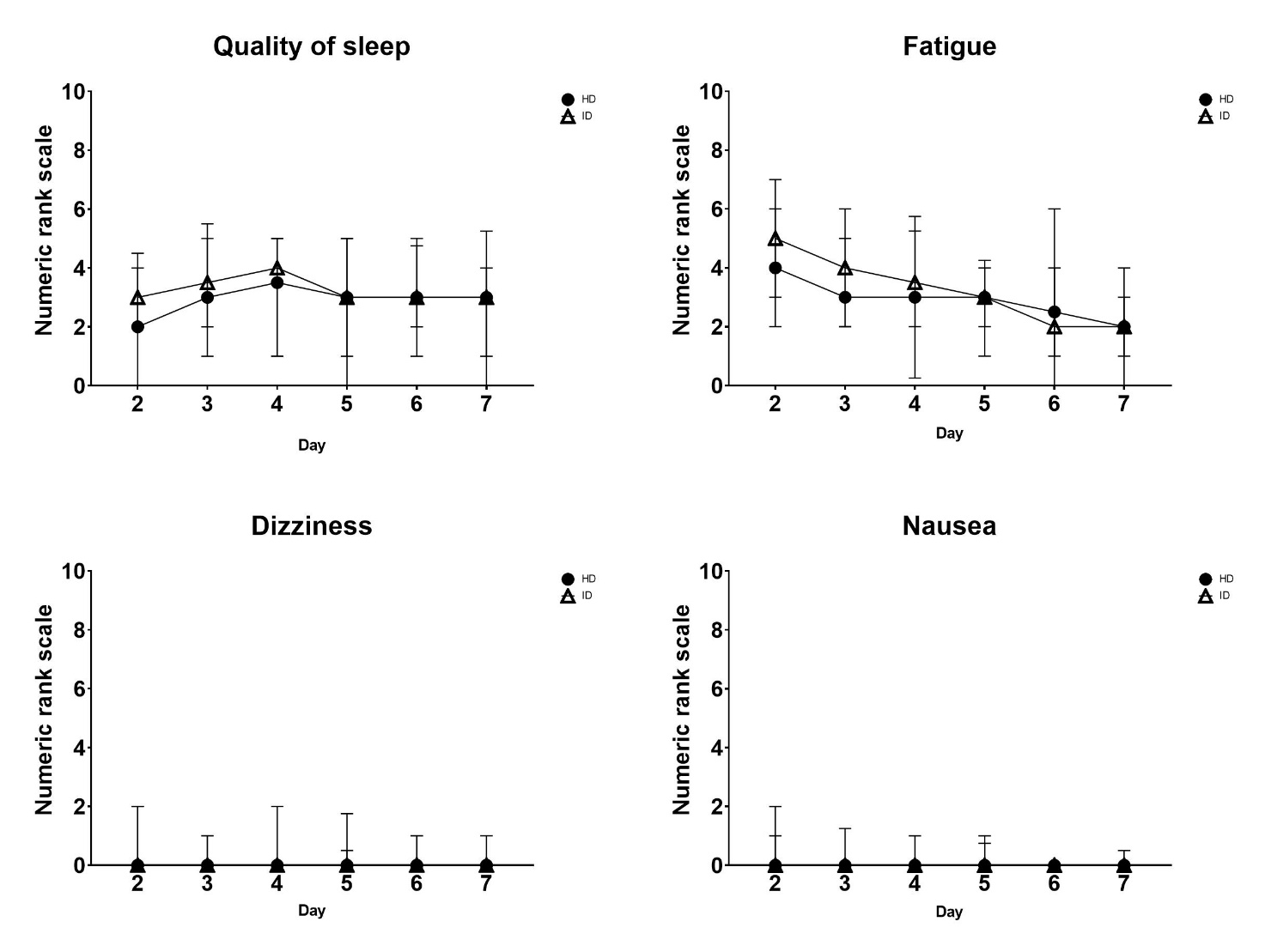


Appendix 4:
Quality of sleep, level of fatigue, dizziness and nausea on a 0-10 point numeric rank scale (NRS) (0 being no troubles and 10 being the worst imaginable) from day 2-7. HD: High dose group, ID: Intermediate dose group.
